# Various Uses of Chloride of Zinc

**Published:** 1875-05

**Authors:** J. E. Nichols

**Affiliations:** Osage, Ia.


A MONTHLY RECORD OF

*Medicine, Surgery and the Collateral Sciences.*

EDITED BY J. ADAMS ALLEN, M.D., LL.D.: AND WALTER HAY, M.D.

---

VOL. XXXII. — MAY, 1875. — NO. 5.

---

### Original Communications.

ARTICLE I.

### VARIOUS USES OF CHLORIDE OF ZINC.

BY J. E. NICHOLS, M.D., OSAGE, IA.

This article is sooner prepared than it was intended, on account of receiving letters of inquiry from professional brethren. To reply fully to their questions, it is necessary to repeat some of the matter of former articles, for which it is hoped pardon will be granted.

For the removal of cancer or other tumors, covered by the integument, rub together *chloride of zinc* and *blood-root* in powder. Use enough of the powdered root to take up the moisture caused by the deliquescence of the zinc, so as to form a plastic, moist dough. It should be firm enough not to spread out beyond the limits desired in its application. Mould it into a sheet, from one line to one-fourth of an inch in thickness, the size and shape of the surface to which it is to be applied, first protecting the surrounding sound parts with adhesive plaster. The paste or dough is kept in place by means of adhesive plaster. The eschar will vary in extent and depth, according to the depth of paste employed. It usually exhausts its strength in forty-eight hours. The eschar will also be somewhat greater in circumference than the surface covered with paste, for which some allowances are to be made. If the first eschar does not embrace the whole of the morbid growth, as is sometimes the case, apply fresh paste to the remnant, protecting the edges of the wound with lint. The agent takes immediate and more extensive effect if there is no intervening integument. In this second application it may be better to employ Dr. Marsden's arsenious mucilage as follows:—℞. arsenious acid, ʒ ij; mucilage gm. acacia ʒ j. M. Ft. a stiff paste. Apply this to not more than one square inch of surface, press lint over it, and when dry, cut off the surplus lint with sharp scissors. This has a more especial election for the morbid structures, not materially affecting the healthy tissues. After removing either of the above pastes at the end of forty-eight hours, and until the eschar comes away, poultices are the best dressings, after which simple cerate, or, in small wounds, court plaster, is all that is required until perfect cicatrization occurs, which usually is very speedily. In my experience, I have never made a wound with chloride of zinc that has not healed quickly and kindly, even when an indolent ulcer was acted upon. It is its quality of arousing healthy inflammation in the parts surrounding the eschar that it produces, that first led me to employ it in other cases than tumors, or cancer, some of which I give below:

Mr. N. Aldrich, astrong laborer, about twenty-eight or thirty years of age, called upon me in March, 1872, with chronic arthritis of the left knee, arising from an injury sustained in 1864, while in the U. S. volunteer service. He had been under the treatment of several good surgeons, only one of whom benefited him any, which he did by the use of a very perfect extension of the knee; but a cure seemed to be impossible. I ordered the reapplication of the extension, and exhausted all the usual palliative modes of treatment. He finally became so anxious to be relieved from his terrible pain, that he was willing to undergo any operation, and even importuned me for several weeks to amputate above the knee. *Erichsen*, in his work on Surgery, advises the use of the actual cautery in some cases of arthritis. He says, "the patient having been anæsthetized, a cauterizing iron heated to a black-red heat, should be rapidly drawn over the diseased articulation in a series of parallel lines, across which an equal number of cross bars are again drawn, so as to char but not destroy the skin," (p. 615 of his second edition). While considering the advisability of employing this, it occurred to me that the zinc paste would answer as well, and would save employing the anæsthetic, and avoid the censure that such an operation would be likely to bring upon a country practitioner if not successful. Drawing the paste out in strings about a line in diameter, I laid them upon the joint, parallel with the limb, and about an inch apart, over the entire swelling, except in the popliteal space. I kept them in place by means of lint and a bandage for about twenty-four hours. They created great swelling and inflammation of the skin and subcutaneous tissues, the pain of which, the patient claimed, was many times compensated for by the absence of the pain in the joint. In places, eschars were formed, and in others, the integument seemed to be only slightly scorched. These healed in a short time, and I employed it the second time in the spaces between the old cicatrices. He was wholly free from pain after the second use of the paste, and the integument and superficial tissues were so drawn down and bound about the articulation that he has had no arthritis to this day. I met him last week, when he informed me that he had not been kept from labor an hour since my treatment, by any pain or lameness of that limb.

July 15, 1874. Mr. H., aged about twenty-two, otherwise healthy, called on me to be treated for syphilis. He had one well defined, indurated chancre upon the glans penis, and two softer ones upon the prepuce. He had been treated with nitrate of silver and acid previously, and objected to their employment now, unless it was absolutely necessary. Protecting the parts about the chancres with lint, I made use of the zinc paste, leaving it upon them about thirty-four hours. The eschars came away in three days, and I was surprised at the rapidity with which the wounds healed. I made use of a building-up treatment, and never had a case do so well before.

Samuel Ryerson, aged sixty-nine years, of good general health, consulted me November 18, 1874, with regard to an ulcer that had been on his face over fifteen years. It involved the inner half of the lower lid of the left eye, nearly all the left side of the nose, and a part of the cheek, as large as a silver dollar. He had another one below the temple, near the outer angle of the left eye, as large as a half dollar, that was not of so long standing as the other. I diagnosed it a *rodent ulcer*, or lupus, or the *noli me tangere.* He had consulted many physicians, both in the East and here, all of whom advised him to let it alone, as to meddle with it might aggravate it. I began the treatment of it by covering the lower half of each ulcer with the zinc paste, using one part zinc to about five parts of the powdered blood-root, allowing it to remain about twelve hours. It caused a thin eschar to form and peel off in about six days, after which I dressed it morning and night, by washing with castile soap suds, and applying pulverized *peruvian bark*, to absorb the secretions from the surfaces of the ulcers.

December 10. The ulcers are healed in over one-third their extent; but as they began to assume an indolent appearance, I applied a still weaker mixture of zinc and blood-root over their entire surfaces, leaving it on only about six hours, as the first application caused more swelling than was desirable. The treatment was continued about the same as before, and resulted in the perfect cure of the smaller ulcer, and the reduction of the larger one to a very small point of ulceration in the inner canthus of the eye.

January 4, 1875. Make a still milder application of paste to the small ulcer in the angle of the eye, allowing it to remain six hours. This produced a very little sloughing from the surface, and only a little inflammation. The remnant of the lower eyelid, which was at first in a bad state of ectropion, is being drawn into a much better shape by the cicatrization than I had supposed possible at the commencement of the treatment.

March 25. There is only a little redness about the greater canthus, and slight ectropium of the lower lid. The puncta lachrimalis, which has opened on the surface of the ulcer throughout its career is perforate, and even the pustules that formerly came upon the face about the ulcers have ceased to appear, and, on the whole, the results are better than I had thought possible. Good diet has constituted the whole of the general treatment.

Another case, learning of my treatment of this, came from a neighboring town for treatment. He has a *rodent ulcer*, embracing both sides and bridge of the nose, with a fistulous opening one-half inch long, by one-fourth inch wide, into the left nostril. This case possessed the characteristic indurated and tuberculated points in the integument about the ulcer, having a bright red and shining appearance. He has now been under treatment about a month, the zinc having been employed as in the other case; and now the ulcer is all healed, excepting a point the size of a little finger nail, and the fistula, which will require a plastic operation to close it.

The case of Nils Anderson, that was reported as under treatment in my last article, terminated by the entire removal of the remnant of morbid growth, and such perfect filling in by granulation that an operation to restore the lip will not be needed. This could not have been secured by the extirpation of the cancer in the usual way, in my opinion.

I have also employed a solution of zinc in acidulated water for the treatment of gleet, and various chronic inflammations of mucous membranes, with good results. In fact, I find it such a valuable agent to stimulate and to create healthy inflammation, that I should be loath to part with it as a therapeutical agent; and I am of the opinion that a fair and careful trial of it will cause others to admire it also. In its use, it should be remembered that, like many good agents, it is powerful to do mischief if carelessly handled.

